# SHEP1 alleviates cardiac ischemia reperfusion injury via targeting G3BP1 to regulate macrophage infiltration and inflammation

**DOI:** 10.1038/s41419-024-07282-5

**Published:** 2024-12-18

**Authors:** Tingwen Gao, Zhenyang Guo, Xinyu Weng, Yikai Cui, Peng Li, Tao Hu, Wei Luo, Zheng Dong, Peng Wei, Yun Cai, Yijing Lu, Rifeng Gao, Hua Li, Xin Zhong, Junbo Ge

**Affiliations:** 1 Department of Cardiology, Zhongshan Hospital, Fudan University, Shanghai Institute of Cardiovascular Diseases, Shanghai, China; 2 Department of Cardiology, Rizhao Heart Hospital, Qingdao University, Rizhao, China; 3 Department of Cardiology, Xijing Hospital, Fourth Military Medical University, Xi’an, Shaanxi China; 4 Department of Cardiology, Shanghai Jiao Tong University Affliated Sixth People’s Hospital, Shanghai, China; 5 Institute of Biomedical Sciences, Fudan University, Shanghai, China; 6 National Clinical Research Center for Interventional Medicine, Shanghai, China; 7 Shanghai Clinical Research Center for Interventional Medicine, Shanghai, China; 8 Key Laboratory of Viral Heart Diseases, National Health Commission, Shanghai, China; 9 Key Laboratory of Viral Heart Diseases, Chinese Academy of Medical Sciences, Shanghai, China

**Keywords:** Integrin signalling, Cell death and immune response

## Abstract

The macrophage-associated inflammation response plays an important role in myocardial ischemia-reperfusion injury (MIRI). SHEP1(SH2 domain-containing Eph receptor-binding protein 1) has been implicated in adhesion and migration of inflammatory cells. However, the role and molecular mechanism of SHEP1 regulating macrophage remains unclear during MIRI. Here, the expression of SHEP1 was increased in macrophages co-cultured with hypoxia-reoxygenated cardiomyocytes and within ischemia-reperfusion injured myocardium at the early stage of injury. Cell migration and inflammation were also enhanced in SHEP1 knock-out macrophages and macrophage-specific deficiency of SHEP1 mice under MIRI, which further led to deteriorated cardiac injury and cardiac function in vivo. Mechanistically, macrophage-derived SHEP1 competitively bound to G3BP1 to suppress inflammation via the MAPK pathway. In addition, administrating inhibitor of G3BP1 could improve cardiac function in macrophage-specific deficiency of SHEP1 mice under MIRI. Our results demonstrate that SHEP1 deficiency in macrophages exacerbates MIRI through G3BP1-dependent signaling pathway. SHEP1-G3BP1 interaction are therefore indispensable for SHEP1 regulated- infiltration and proinflammatory responses of macrophages, which provided a potential and clinically significant therapeutic target for MIRI.

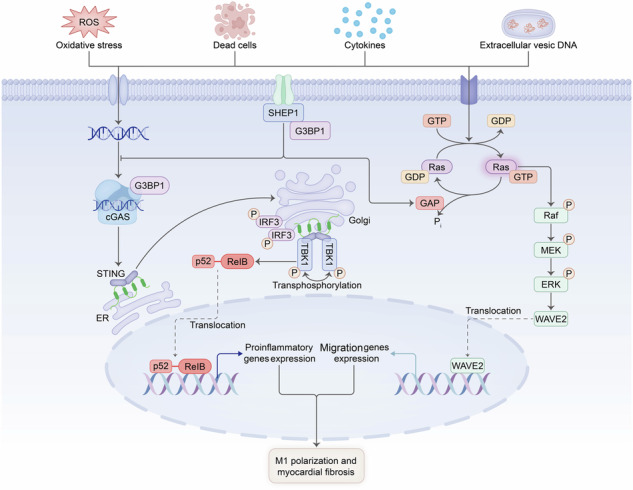

## Introduction

Acute myocardial infarction (AMI) is a major threat to human health worldwide. Revascularization therapy significantly improves the prognosis of AMI, but death rates due to secondary reperfusion injury remain persistently high. Accumulating evidence suggests that inflammatory mediators released from the innate and adaptive immune responses contribute to the progression of myocardial ischemia-reperfusion injury (MIRI) [1, 2]. MIRI induces a vigorous immune response, augmented by the generation and release of various inflammatory cytokines and chemokines that accelerate peripheral monocyte mobilization and infiltration to exacerbate myocardial injury [3, 4]. This ultimately leads to adverse outcomes including infarct expansion, left ventricular (LV) systolic dysfunction, and LV dilation [5]. Macrophages involved in MIRI exhibit functional heterogeneity, with pro-inflammatory macrophages infiltrating initially to clear the irreversibly damaged tissues, followed by a reparative phase of anti-inflammatory macrophages that drives the resolution of inflammation, tissue repair, and coronary angiogenesis [6]. Both migration and differentiation play vital roles in inflammatory processes. However, little is known about the initial mechanisms by which monocytes are recruited to the sites of injury, the diversity of monocyte-derived macrophages, or the instructive cues that specify their fate.

To explore the regulators that modulate monocyte recruitment and fate specification during MIRI, we previously sequenced transcriptomes of peripheral blood mononuclear cells derived from 10 patients assigned to percutaneous coronary intervention due to acute ST‐segment elevation myocardial infarction at successive timepoints launching around the intervention. The results demonstrated that the mRNA levels of SHEP1 were significantly associated with revascularization [7], suggesting that it may play a role in MIRI. SHEP1, encoded by the *SH2D3C* gene, is an Src homology 2 (SH2) -containing NSP scaffold that mediates protein-protein interactions through conserved modular domains [8]. Its architectures include an amino-terminal SH2 domain that binds to phosphorylated tyrosine residues, followed by a proline/serine-rich region, and a conserved carboxyl-terminal domain with homology to CDC25-related guanine nucleotide exchange factors (GEFs), which promote guanine nucleotide exchange on Ras family GTPases. Although the GEF domain of SHEP1 binds to several Ras proteins, it appears to lack enzymatic activity [9]. It has been shown that NSP family members affect the migration and adhesion of T cells and B cells by regulation of cell signaling [10, 11]. In addition, they act as essential regulators of monocyte migration during atherosclerosis development [12]. However, whether and how SHEP1 participates in macrophage function during MIRI remains unclear.

To ascertain the role of SHEP1 in macrophages during MIRI, we examined cardiac repair in mice genetically engineered to lack SHEP1 in macrophages. Our findings reveal that a deficiency of SHEP1 in macrophages antagonizes optimal cardiac rehabilitation by promoting macrophage infiltration while fostering pro-inflammatory responses, thus providing an attractive new approach to preventing adverse events in MIRI.

## Materials and methods

### Isolation and culture of adult mouse primary cardiomyocytes

Mouse cardiomyocytes were isolated from WT mice using Liberase enzymatic digestion as previously described [13].

### Cell co-culturing

The cell co-culturing system was performed using Corning Transwell chambers (0.4 µm pores, 6.5 mm diameter) where primary cardiomyocytes from adult wild-type C57 mouse were hypoxia-injured (in low-glucose DMEM without FBS, incubated in a 37 °C air-tight incubator with 0.1% O_2_, 5% CO_2_ balanced by N2 for 6 h) in the upper well, subsequently reoxygenated (back to high-glucose DMEM with 10% FBS), and concurrently inserted into lower well laid with RAW264.7 followed by incremental periods of co-culturing.

### RNA isolation and quantitative PCR (qPCR)

Total RNA was extracted with TRIZOL reagent (93289, Sigma) according to the manufacturer’s protocol and quantified using a Nanodrop 2000. Total RNAs (1000 ng) were used to perform the reverse transcription with the PrimeScript RT reagent kit (Takara, DRR037A). To determine relative mRNA abundance, qPCR was performed with SYBR Green Master Mix (Yeason) on an ABI StepOnePlus system according to the manufacturer’s protocol. Data were analyzed with StepOnePlus software. The primers were synthesized from Tsingke Biotechnology and sequence information is provided in Supplementary Table 1. Mouse 18S was used for normalization. The experiment was repeated three times.

For RNA-seq, after RNA extraction and quantification, Oliago(dT)-attached magnetic beads were used to purify mRNA. Purified mRNA was fragmented into small pieces with fragment buffer at an appropriate temperature. Then First-strand cDNA was generated using random hexamer-primed reverse transcription, followed by second-strand cDNA synthesis. Afterward, A-Tailing Mix and RNA Index Adapters were added by incubating to end repair. The cDNA fragments obtained from the previous step were amplified by PCR, and products were purified by Ampure XP Beads, then dissolved in EB solution. The product was validated on the Agilent Technologies 2100 bioanalyzer for quality control. The double-stranded PCR products from the previous step were heated denatured and circularized by the splint oligo sequence to get the final library. The single-strand circle DNA (ssCir DNA) was formatted as the final library. The final library was amplified with phi29 to make a DNA nanoball (DNB) which had more than 300 copies of one molecular. DNBs were loaded into the patterned nanoarray and single end 50 bases reads were generated on the BGIseq500 platform (BGI-Shenzhen, China). The sequencing data was filtered with SOAPnuke (v1.5.2) by (1) Removing reads containing sequencing adapter; (2) Removing reads whose low-quality base ratio (base quality less than or equal to 5) is more than 20%; (3) Removing reads whose unknown base (‘N’ base) ratio is more than 5%, afterward clean reads were obtained and stored in FASTQ format. The clean reads were mapped to the reference genome using HISAT2 (v2.0.4). Bowtie2 (v2.2.5) was applied to align the clean reads to the reference coding gene set, then the expression level of the gene was calculated by RSEM (v1.2.12). The heatmap was drawn by heatmap (v1.0.8) according to the gene expression in different samples. Essentially, differential expression analysis was performed using the DESeq2 (v1.4.5) with Qvalue ≤ 0. 05. To take an insight into the change of phenotype, GO (http://www.geneontology.org/) and KEGG(https://www.kegg.jp/) enrichment analysis of annotated different expressed genes was performed by Phyper (https://en.wikipedia.org/wiki/Hypergeometric_distribution) based on Hypergeometric test. The significant levels of terms and pathways were corrected by Q value with a rigorous threshold (Qvalue ≤ 0. 05) by Bonferroni.

### Protein sample preparation, IP-MS, and Western blotting assay

Total proteins were extracted in RIPA lysis buffer (Beyotime, China) supplemented with a Complete Mini protease inhibitor cocktail (Roche, 11836153001). The protein concentration was determined by the BCA protein assay kit (Bio-Rad, 5000006JA).

For immunoprecipitation (IP), the Pierce Classic IP Kit (Thermo Scientific™, 26146) was utilized according to the manufacturer’s protocol.

For identification of shep1-interacting proteins, liquid chromatography-tandem mass spectrometry (LC-MS/MS) analysis was performed. Immunoprecipitates were separated with SDS–PAGE and the gel was stained with Coomassie brilliant blue for protein band visualizing. The entire lane was cut into 1 cm gel slices and subjected to in-gel digestion. The mass spectrometry analysis was conducted by PTM-BIO (China). The ranking of the identified proteins was based on the peptide coverage of each identification. The related proteins were selected and subjected to further validation by immunoprecipitation and immunoblot. This study focused on G3BP1.

After quantified, protein samples (10–20 μg) were loaded on SDS-polyacrylamide gels for electrophoresis and then transferred to PVDF membranes. After blocking with 5% BSA for 1 h, the membranes were incubated with primary antibodies overnight at 4 °C. After the subsequent washes, the membranes were incubated with horseradish peroxidase (HRP)-conjugated secondary antibodies (Weiao Biological Company, China) at room temperature for 1 h. The blots were visualized and detected by Chemiluminescence Reaction (LuminataTM Forte, Millipore, USA) and ChemiDocTM Imaging System (Bio-Rad, CA, US). The density of the protein blots was analyzed by ImageJ (Version 1.53c, NIH, USA). Detailed information on the primary antibodies used in immunoblotting, including source, catalog number, and dilution, is listed in Supplementary Table 2. We also provide full scans of all the blots as Supplementary Dataset.

### Migration wound healing

Migration assays were performed using Corning Transwell chambers (8 µm pores, 6.5 mm diameter, USA). Primary cardiomyocytes were isolated from adult wild-type C57 mice, laid in the lower wells, and exposed to H/R injury. After reoxygenation, RAW264.7 cells were seeded in the upper chambers containing FBS-free DMEM. After 24 h of co-culture, cells that traversed through the membrane were fixed with 4% PFA for 30 min and stained with 0.05% crystal violet solution (Solarbio, China) for 20 min.

A wound-healing assay was conducted in 6-well plates by scratching the cell monolayer with a 200 µl pipette tip. Cells were washed with PBS twice and cultured in conditional media of H/R cardiomyocytes. Images were collected at the beginning and 24 h after scratching. The wound-healing speed was calculated as the ratio of the healed areas at 24 h to the original injured areas.

### RNA interference

RAW264.7 cells were transfected with siRNAs by Lipofectamine™ 3000 transfection reagent (Invitrogen™, L3000150) for 48 h before stimulation according to the manufacturer’s instruction. The siRNAs used were as follows: si-cGAS (5′-CGGCAGCUAUUAUGAACAU-3′), si-G3BP1(5′-CCCTATGGAAATCATTCCT-3′).

### Mice

Mice were fed with a standard rodent chow diet and housed in micro isolator cages in a special pathogen-free facility. The number of animals in each experimental group is indicated in the tables or figure legends and was set up according to previous research. This study and all animal procedures conformed to the Guide for the Care and Use of Laboratory Animals, published by the National Institutes of Health (NIH publication NO. 85–23, revised 1996), and were approved by the Animal Care and Use Committee of Fudan University.

Wild-type (WT) C57BL6/J mice (male, 14–16 weeks old; SLRC Laboratory Animal, Shanghai, China) were used in this study and randomly divided into groups.

Myeloid-specific SHEP1-deficient mice were generated by crossing SHEP1^fl/fl^ mice with LysM-Cre mice and are designated as LysM Cre^+^ SHEP1^fl/fl^ (CKO) in this paper (constructed by Cyagen Biosciences Inc. China). For experiments described in this paper, adult male mice (14–16 weeks of age) were used.

EGCG administration: Mice were intraperitoneally injected with EGCG (40 mg/kg) right after reperfusion, and afterward once a day for 3 days.

### Cardiac I/R injury model

Mice underwent surgical cardiac I/R injury as described previously [14]. Briefly, the mice were anesthetized with 2% isoflurane inhalation using an isoflurane delivery system. Subsequently, a small skin cut (1.2 cm) was made over the left chest, and the left anterior descending coronary artery (LAD) was ligated via a slipknot using 6–0 silk for 45 min before the slipknot was gently loosened to induce reperfusion injury. The suture was passed 2–3 mm below the tip of the left auricle. Sham-operated animals underwent the same procedure without tying the slipknot.

### Triphenyltetrazolium chloride (TTC) staining

TTC staining was conducted for evaluation of cardiac infarction size. After the mice were anesthetized with 2% isoflurane, the slipknot was tied again, and 1% Evans blue (w/v, Sigma, USA) was injected into the aortic root to perfuse the left ventricle. Then, the heart was rapidly excised, cut into 1 mm slices, and incubated with 1% TTC solution (Sigma, USA) at 37 °C for 10 min. Consecutive slices were scanned by a white light scanner (Canon, Japan). LV area, AAR, and infarct area were determined by computerized planimetry and comprehensively analyzed in serial sections of each mouse using ImageJ software. The infarction degree was calculated as the ratio of the infarction area to the AAR.

### Transthoracic echocardiography

Cardiac function was assessed with a Vevo2100 Ultrasound system (VisualSonics, Toronto, Canada). Mice were mildly anesthetized with 0.5% isoflurane and placed on a heated ECG platform. The left ventricle and the aortic outflow tract were observed under the two-dimensional B-Mode, and the sample line was placed at the maximum cross-section of the left ventricle to guide the recording of serial M-Mode echocardiographic images. The left ventricular end-systolic volume (LVESV), left ventricular end-diastolic volume (LVEDV), fractional shortening (FS), left ventricular end-systolic diameter (LVESD), and left ventricular end-diastolic diameter (LVEDD) were measured from at least three distinct frames for each mouse, and the average was used for data analysis. The sonographer was blinded to the group allocation.

### IHC and immunofluorescence staining

Analysis was performed using paraffin-embedded sections of the heart tissue samples fixed with 4% paraformaldehyde. All tissue samples were characterized using hematoxylin and eosin (H&E) staining before further immunostaining. After the inhibition of endogenous peroxidase activity, the sections were incubated with primary anti-SHEP1 (sc-100792, Santa Cruz) at 4 °C overnight. Following the incubation, a Vectastain Elite ABC kit (Vector Laboratories, Burlingame, CA, USA) was used according to the manufacturer’s instructions. After the visualization with 3,3′-diaminobenzidine, the sections were counterstained with hematoxylin. As for immunofluorescence analysis, after deparaffinization, heat-mediated antigen retrieval in citrate buffer pH 6.0, and blocking with 5% horse serum at room temperature for 30 min, samples were incubated with primary antibodies overnight at 4 °C. The primary antibodies anti-SHEP1 (Santa Cruz, 1:100, sc-100792) and anti-F4/80 (AbD Serotec, 1:200, MCA497GA) were utilized for these experiments. Signals were visualized with Alexa fluorescence-conjugated secondary antibodies (Invitrogen, Carlsbad, CA, USA). TUNEL staining was performed with an In Situ Apoptosis Detection kit (Yeasen, Shanghai, China), according to the manufacturer’s instructions. Cell nuclei were stained with DAPI. Five fields or more in >3 different sections/animals were examined by a technician who was blinded to the treatment groups. Images were obtained using an Olympus microscope and quantified using Image J. For each heart sample, at least five random fields were measured.

### Cell extract, flow cytometry, and magnetic beads sorting

The hearts were dissected, carefully cut into small pieces with fine scissors, and enzymatically digested with type II collagenase (1.5 mg mL^−1^, Worthington Biochemical, Lakewood, NJ, USA), elastase (0.25 mg mL^−1^, Worthington Biochemical), and DNase I (0.5 mg mL^−1^, Worthington Biochemical) for 1 h at 37 °C. Following digestion, the tissues were passed through 70-μm cell strainers, washed, and stained with antibodies for fluorescence-activated cell sorter (FACS) analysis. To block the non-specific binding of antibodies to Fcγ receptors, isolated cells were incubated first with anti-CD16/32 antibody (BD Biosciences, #553142) at 4 °C for 5 min. The primary antibodies against the following were obtained from BD Biosciences (Franklin Lakes, NJ, USA): CD45.2 (#564616), CD11b (#552850), Ly-6G (#560599), Ly6C (#563011), CD64 (Biolegend, #139309), CD206 (Thermo, 17-2061-82). The obtained results are expressed as cell number/µg tissue. Flow cytometric analysis was performed using a FACSAria™ flow cytometer (BD Biosciences), and the obtained data were analyzed with FlowJo 7.6.1 software (Tree Star, Ashland, OR, USA).

Magnetic cell sorting was performed using QuadroMACS (Miltenyi Biotec, 130-091-051) according to the manufacturer’s instructions, Anti-f4/80 MicroBeads (Miltenyi Biotec, 130-110-443) were utilized for separating macrophages.

### Statistical analysis

Data were collected and expressed as average ±standard error of the mean (SEM). *T-*test were conducted when comparing 2 independent groups with the normalized distribution of values. For *n* = 3–4 per group, Mann-Whitney tests were used. ANOVA-test or Kruskal-Wallis (with low numbers) were carried out when comparing more than 2 groups. For two groups and multiple time points, a two-way ANOVA was used. Statistical analysis was conducted using GraphPad Prism 8.0 (GraphPad Prism Software Inc., San Diego, CA, USA).

## Results

### SHEP1 selectively expressed in macrophages co-cultured with H/R cardiomyocytes fluctuates over time

**Fig. 1:**
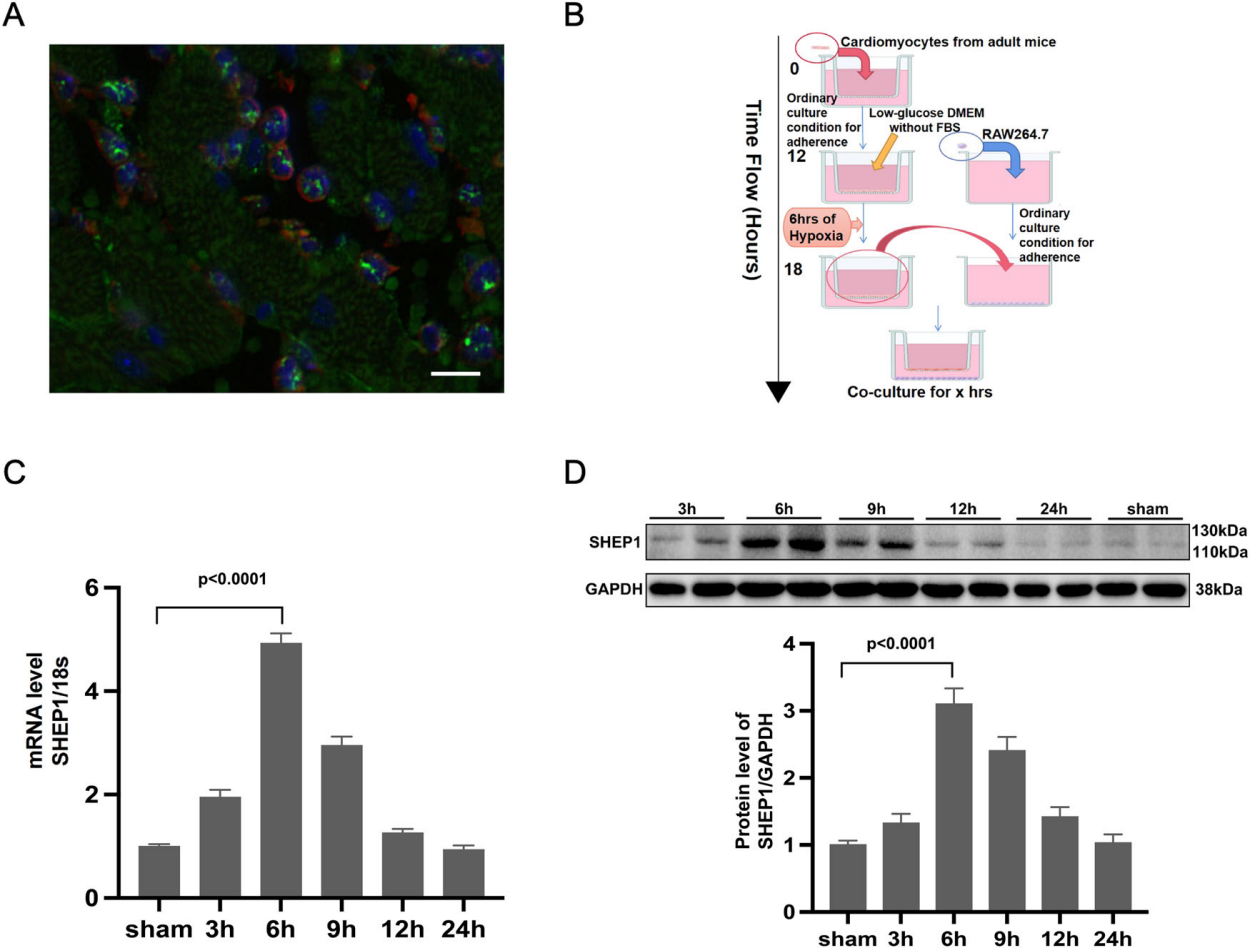
SHEP1 selectively expressed in macrophages co-cultured with H/R cardiomyocytes fluctuates over time. **A** Representative dual-immunofluorescence staining of SHEP1(green), F4/80(red), and nuclei (4,6-diamidino-2-phenylindole, blue) in mice hearts 1 day after IR. Scale bar, 10 μm. **B** Flow chart of the co-culture system. SHEP1 mRNA levels (**C**) and protein levels **(D)** in RAW264.7 co-cultured with hypoxia-reoxygenated cardiomyocytes for different time periods (*n* = 3 sets of cells). Data are presented as mean ± SEM.

We first co-stained heart tissue slices from wild-type C57BL6 mice that had undergone cardiac IR operation with fluorescent-labeled antibodies for macrophages (F4/80, red) and SHEP1 (green) to investigate whether SHEP1 selectively expressed in macrophages. And DAPI (blue) was used to stain the nuclei. The results demonstrated that SHEP1 was specifically localized in the cytoplasm of macrophages in merged confocal images (Fig. 1A).

To mimics microenvironment in vivo under the condition of MIRI, we established co-culture systems with transwells between macrophages and hypoxia-reoxygenated (H/R) cardiomyocytes (flow chart shown in Fig. 1B) to investigate the evolution of SHEP1 expression in RAW264.7 cells over time. The results showed that SHEP1 mRNA and protein levels peaked at 6 h after co-culture, then both gradually declined to baseline level within 24 h (Fig. 1C, D).

### SHEP1 deletion in RAW264.7 co-cultured with H/R cardiomyocytes enhances macrophages migration and inflammation in vitro

**Fig. 2:**
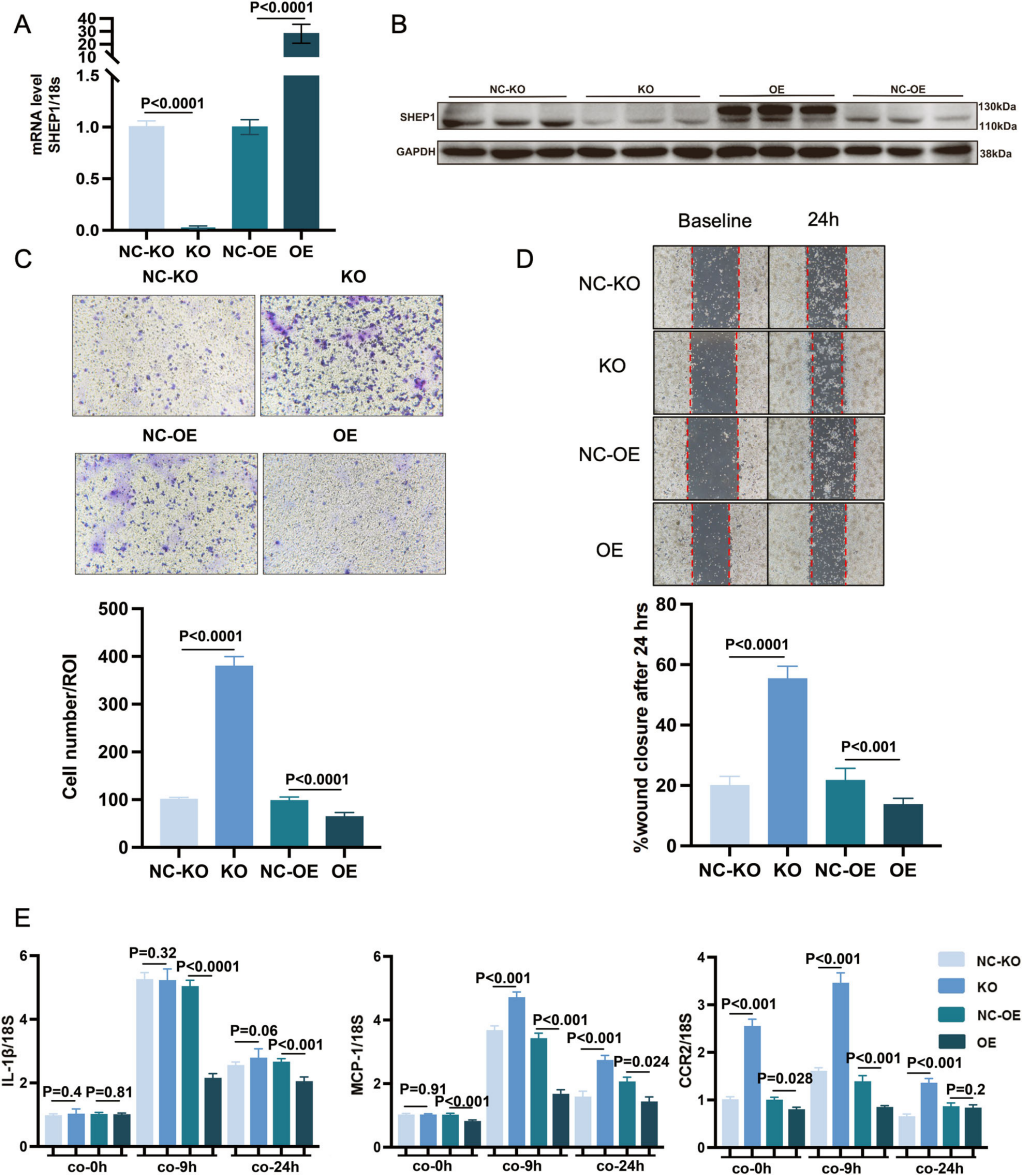
SHEP1 deletion in RAW264.7 co-cultured with H/R cardiomyocytes enhances macrophages migration and inflammation in vitro. **A** SHEP1 mRNA levels in SHEP1 knocked-out and over-expressed RAW264.7 as well as their respective negative controls (NC-KO and NC-OE) analyzed by RT-qPCR. **B** SHEP1 protein levels in SHEP1 knocked-out and over-expressed RAW264.7 as well as their respective negative controls (NC-KO and NC-OE) analyzed by western-blot. **C** Representative images of transwell migration assay in which upper-well RAW264.7 migrated towards lower-well hypoxia-reoxygenated (H/R) cardiomyocytes. Cell numbers were calculated and compared (*n* = 6). **D** Representative images of cell scratch assay in which RAW264.7 monolayers were scratched and afterwards exposed to the supernatant derived from H/R cardiomyocytes. Cell numbers were calculated and compared (*n* = 6). **E** mRNA levels of IL-1β, MCP-1, and CCR2 in SHEP1 knocked-out/over-expressed RAW264.7 and their respective negative controls (NC-KO and NC-OE) co-cultured with H/R primary cardiomyocytes from adult mouse for 9 or 24 h in our co-culture system described above (*n* = 3 sets of cells). All data are presented as mean ± SEM.

To observe the effect of SHEP1 on the biological function of monocyte in vitro, we thereupon constructed SHEP1 knock-out (KO) and over-expressed (OE) RAW264.7 monoclonal cell lines for verification by qPCR and western-blot (Fig. 2A, B**)**. By the above transwell co-culture systems, the migration of SHEP1-KO RAW264.7 group was significantly promoted than that of NC-KO group, whereas it showed the opposite trend between the SHEP1-OE group and NC-OE group (Fig. 2C). And another result with the same biological characteristics was confirmed by wound healing in vitro among these 4 groups (Fig. 2D).

Simultaneously, the effect of SHEP1 regulating inflammation in macrophage was also investigated through using the co-culture system described above. qRT-PCR showed that the pro-inflammatory cytokine IL-1β, chemokine MCP-1 and CCR2 were obviously upregulated in the SHEP1-KO RAW264.7 group (lower-chamber), while down-regulated in the SHEP1-OE group, compared with their corresponding control groups. Unlike that CCR2 showed significant differences at the baseline level, the differences of IL-1β and MCP-1 were gradually occurred after co-culture stimulation, which might mainly be due to that IL-1β and MCP-1 are inflammatory factors produced by macrophages in response to the stimulation of an inflammatory environment, whereas CCR2 represents the susceptibility of macrophages to chemokines (Fig. 2E).

### SHEP1 regulates macrophages activities primarily through the MAPK signaling pathway

**Fig. 3:**
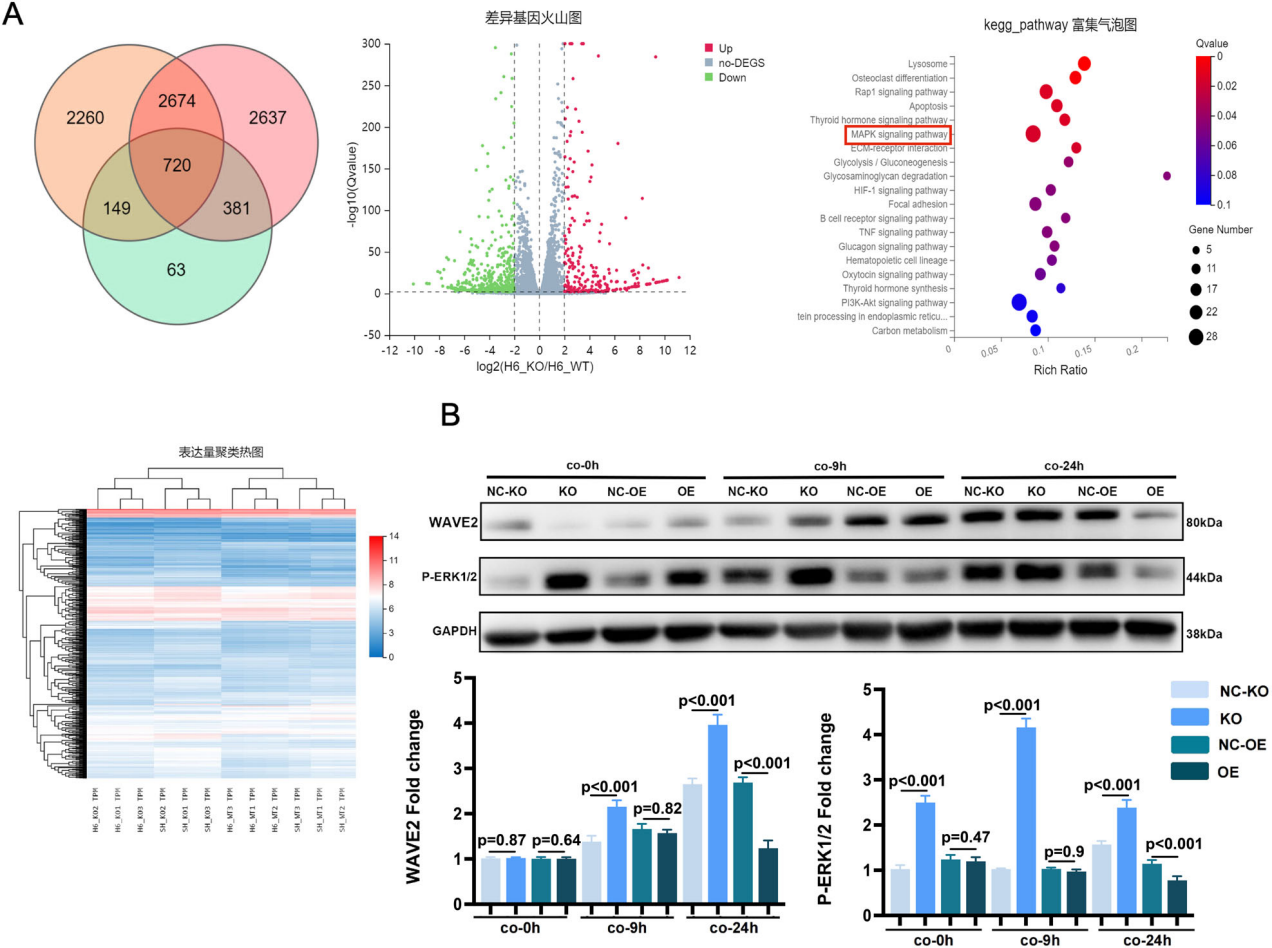
SHEP1 regulates macrophages activities primarily through the MAPK signaling pathway. **A** RNA-sequencing (RNA-seq) for total RNA extracted from RAW264.7 cells (SHEP1 KO and WT) co-cultured for 0 h (SH) or 6 h (H6) with H/R primary cardiomyocytes isolated from adult mice. VENN, volcano blots, KEGG enrichment analysis and Heatmap are displayed. **B** WAVE2 and p-ERK1/2 protein levels in SHEP1 knocked-out (KO)/over-expressed (OE) RAW264.7 and their respective negative controls (NC-KO and NC-OE) co-cultured with H/R primary cardiomyocytes from adult mice for 9 or 24 h (*n* = 3 sets of cells). All data are presented as mean ± SEM.

To elucidate the molecular mechanisms of SHEP1 in various cell activities, we carried out RNA-sequencing (RNA-seq) for total RNA extracted from RAW264.7 cells (SHEP1-KO group and control group, respectively) co-cultured for 0 h or 6 h with H/R primary cardiomyocytes isolated from adult wild-type C57 mice. The transcriptome-wide role of SHEP1 in RAW264.7 cells was evaluated in the co-culture system to identify the key pathways that may be affected by the loss of SHEP1. In total, 720 differential genes were obtained from a Venn diagram analysis of different cell groups for the same co-culture duration and different co-culture durations from the same cell group (Fig. 3A). Subsequently, KEGG pathways analysis among these differential genes indicated that the primary RAS-MAPK-ERK pathway was identified from top 20 pathways and partially involved in cell migration (Fig. 3A).

To illuminate the regulatory role of SHEP1 in RAS-MAPK-ERK signaling pathways, western blotting was used to detect the expression of ERK phosphorylation and its downstream protein WAVE2. The results showed that it was a negative regulation of this pathway by SHEP1, with increased expression of ERK phosphorylation and WAVE2 in SHEP1-KO RAW264.7 cells, while decreased expression in the SHEP1-OE group, when they co-cultured with H/R cardiomyocytes (Fig. 3B).

### SHEP1 targets G3BP1 to attenuate macrophages migration and inflammation via MAPK signaling pathway

**Fig. 4:**
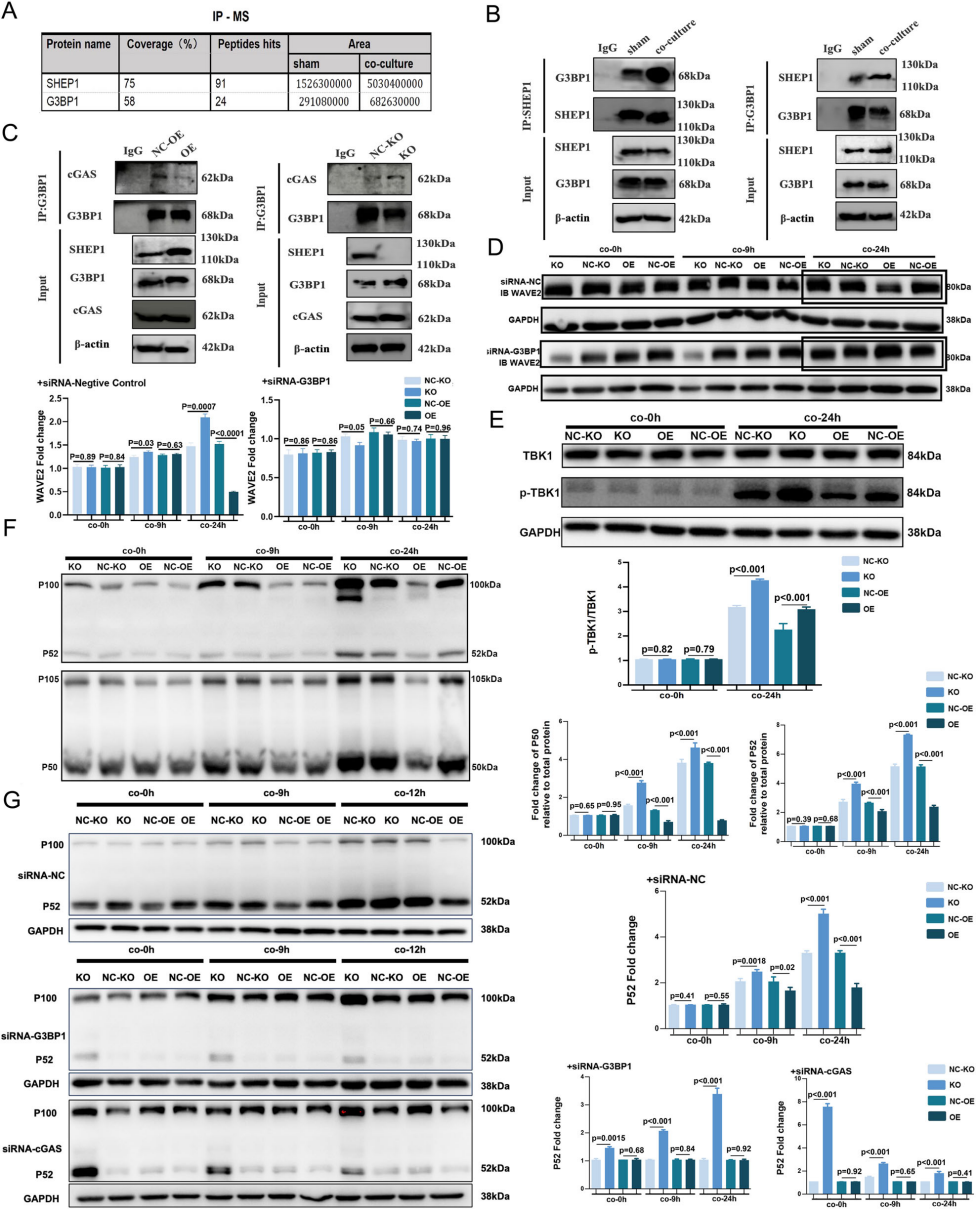
SHEP1 targets G3BP1 to attenuate macrophages migration and inflammation via MAPK signaling pathway. **A** Mass spectrometry analysis of SHEP1 immunoprecipitates. Selected peptide hits and coverage are shown. **B** RAW264.7 co-cultured with H/R primary cardiomyocytes or sham were subjected to co-immunoprecipitation (co-IP) respectively with anti-SHEP1 and anti-G3BP1 antibodies. **C** SHEP1 knocked-out (KO)/over-expressed (OE) RAW264.7 cells and their respective negative controls (NC-KO and NC-OE) cells co-cultured with H/R primary cardiomyocytes or sham were subjected to co-immunoprecipitation (co-IP) respectively with anti-G3BP1 antibodies. **D** SHEP1 KO, OE and NC-KO, NC-OE RAW264.7 cells with or without G3BP1 deficiency were co-cultured with H/R primary cardiomyocytes for 9 or 24 h. Protein levels of WAVE2 were analyzed and compared (*n* = 3 sets of cells). **E** TBK1 and p-TBK1 protein levels in SHEP1 knocked-out (KO)/over-expressed (OE) RAW264.7 and their respective negative controls (NC-KO and NC-OE) co-cultured with H/R primary cardiomyocytes from adult mice for 9 or 24 h (*n* = 3 sets of cells). **F** P100/52 (NFκB2) and P105/50 (NFκB1) protein levels in SHEP1 knocked-out (KO)/over-expressed (OE) RAW264.7 and their respective negative controls (NC-KO and NC-OE) co-cultured with H/R primary cardiomyocytes from adult mice for 9 or 24 h (*n* = 3 sets of cells). **G** SHEP1 knocked-out (KO)/over-expressed (OE) RAW264.7 and their respective negative controls (NC-KO and NC-OE) with or without G3BP1 or cGAS deficiency were co-cultured with H/R primary cardiomyocytes for 9 or 24 h. Protein levels of P100/52 (NFκB2) were analyzed and compared (*n* = 3 sets of cells). All data are presented as mean ± SEM.

To further explore interactive proteins targeted by SHEP1, Co-IP mass spectrometry and competition assay were used to elucidate these potential molecular cluster in RAW264.7 cells and their possible regulation of the RAS-MAPK-ERK pathway. Firstly, differentially expressed proteins binding to SHEP1 in RAW264.7 cells between co-cultured with H/R cardiomyocytes for 9 h and the normoxia group was analyzed (Fig. 4A). Among these differential proteins, Ras-GTPase-activating protein SH3 domain binding protein1(G3BP1) was an optimal candidate since it could bind to the SH3 domain of Ras-GTPase-activating protein (RasGAP), which might regulate RAS-MAPK-ERK signaling pathway [15]. Subsequently, the direct interaction between SHEP1 and G3BP1 was confirmed through Co-IP experiment when RAW264.7 cells were co-cultured with H/R cardiomyocytes (Fig. 4B). Previous studies have shown that G3BP1could promote the activation of cGAS through combining with cGAS [16, 17]. Therefore, in order to explore whether the binding of SHEP1 and G3BP1 affects the binding of G3BP1and cGAS, we examined the binding capacity of G3BP1and cGAS in RAW264.7 cells with SHEP1 overexpression and knockout. The results showed that H/R cardiomyocytes significantly promoted the binding of G3BP1 and cGAS in SHEP1-KO cells, while the opposite was true in SHEP1-OE cells (Fig. 4C).

Secondly, via gene regulation of targeting protein G3BP1, the effect of SHEP1 on expression of its downstream proteins in MAPK signaling pathway was also observed. The results indicated that siRNA-assisted knockdown of G3BP1 abrogated increased expression of WAVE2 in SHEP1-KO RAW264.7 group and decreased expression of WAVE2 in the SHEP1-OE group, which showed that through G3BP1, SHEP1 had a regulatory effect on the RAS-MAPK-ERK pathway, thereby affecting cell migration (Fig. 4D).

Thirdly, as mentioned above, G3BP1 has been revealed to promote the activation of cGAS via DNA binding, and subsequently to activate NF-κB in previous studies [16, 17]. To clarify that SHEP1 suppresses the expression of inflammatory factors by competitive binding with G3BP1 to interrupt the G3BP1-cGAS interaction, western blotting was applied to detect the expression of TBK1 phosphorylation and proteins of NF-κB pathway. The results showed that SHEP1 also could suppress the activation of cGAS, with evidence of the repression of TBK1 phosphorylation (Fig. 4E) and the activation of proteins of NF-κB pathway (Fig. 4F) in SHEP1-KO RAW264.7 group, while an opposite trend was observed in the SHEP1-OE group. Further, blockage of either G3BP1 or cGAS could abolish these effects of SHEP1 on proteins related to NF-κB (Fig. 4G).

### The expression of SHEP1 shows sequential characteristic under the condition of MIRI in vivo

**Fig. 5:**
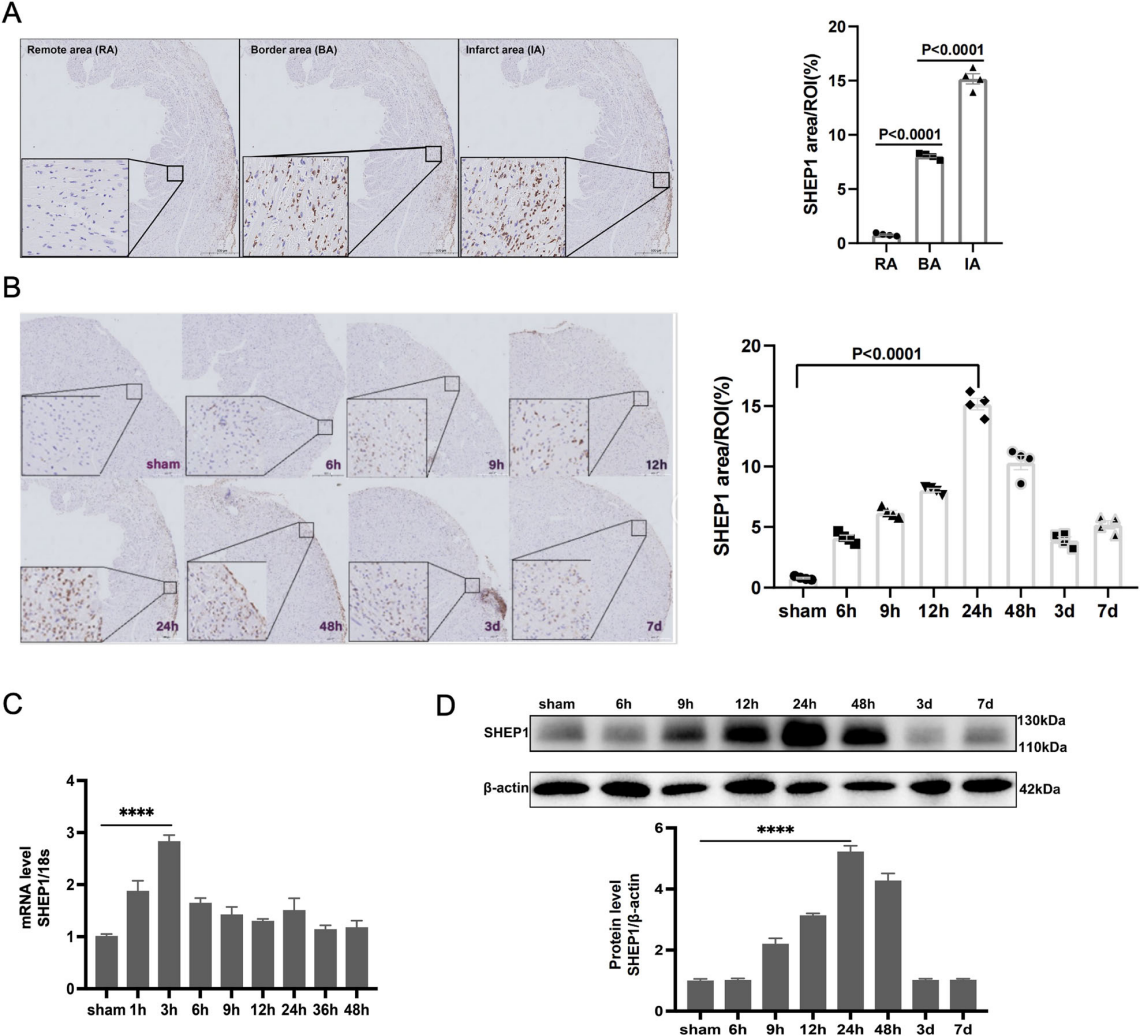
The expression of SHEP1 shows sequential characteristic under the condition of MIRI in vivo. **A** Representative immunohistochemical analyses of SHEP1 at different time points after IR. Scale bar, 500 μm. SHEP1 expression were quantified and compared (*n* = 4). **B** Representative immunohistochemical analyses of SHEP1 in remote area, border area, and infarct area on day 1 after IR. Scale bar, 500 μm. SHEP1 expression were quantified and compared (*n* = 4). **C** S SHEP1 mRNA levels were analyzed by RT-qPCR at different time points after MIRI (*n* = 4). **D** SHEP1 protein levels in myocardium were examined at different time points after MIRI by western blot with SHEP1 antibody, β-actin was used as loading control (*n* = 4). All data are presented as mean ± SEM.

Immunohistochemistry showed that SHEP1 was expressed mainly in the infarct and border areas after MIRI, with low expression in the non-infarct areas (Fig. 5A). Through continuous observation of expression of SHEP1 in histological sections for 7 days, SHEP1 area was found to reach its peak at 24 h after MIRI and afterward experienced a gradual decline (Fig. 5B). To elucidate the expression of SHEP1 in cardiac tissue under the condition of MIRI at different reperfusion time points, we checked the gene expression levels and protein abundance of SHEP1 in the myocardium. The results uncovered that mRNA level of SHEP1 increased as early as 3 h after MIRI and peaked at 6 h, while protein accumulation progressively increased from 9 h to 24 h and subsequently declined (Fig. 5C, D). These data in vivo showed the similar tendency with the western blot results of cells in vitro.

### Macrophage-specific deficiency of SHEP1 deteriorated cardiac function and cardiac injury under MIRI in vivo

**Fig. 6:**
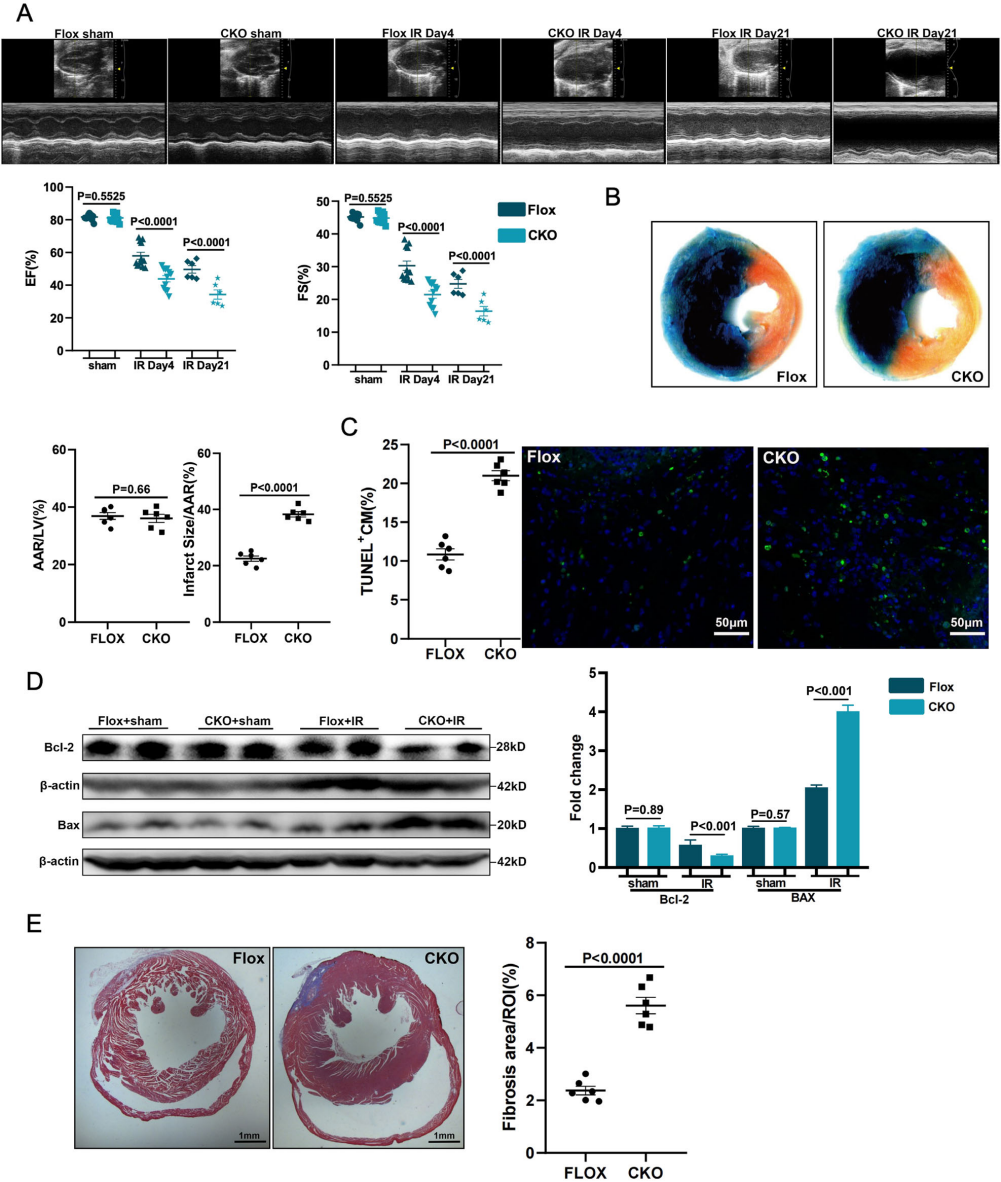
Macrophage-specific deficiency of SHEP1 deteriorated cardiac function and cardiac injury under MIRI in vivo. **A** Representative cardiac echocardiography images at 3 and 21 days after MIRI with quantification of percentage ejection fraction (% EF) and percentage fractional shortening (% FS) (*n* = 12) (**B**) Representative heart sections from *LysMCre*^*+*^*Shep1*^*fl/fl*^ or *Shep1*^*fl/fl*^ mice stained with Evans-blue and 2,3,5-triphenyltetrazolium chloride (TTC) at 1 day after MIRI to delineate the area at risk (AAR) and the infarcted region. The ratios of AAR/LV and infarct area/AAR were compared (*n* = 6). **C** Representative photomicrographs of terminal deoxynucleotidyl transferase–mediated deoxyuridinetriphate nick-end labeling (TUNEL) and nuclear (DAPI) staining of myocardium obtained 1day after MIRI. TUNEL-positive cell number per ROI were compared (*n* = 6). **D** Representative Western blot analysis showing the protein levels of Bax and Bcl-2 in *LysMCre*^*+*^*Shep1*^*fl/fl*^ and *Shep1*^*fl/fl*^ mice hearts at 1 day after MIRI or sham operation (*n* = 6). **E** Representative Masson trichrome staining of cardiac tissue obtained from *LysMCre*^*+*^*Shep1*^*fl/fl*^ and *Shep1*^*fl/fl*^ mice at day 21 after MIRI. Quantitative analysis of fibrotic areas (*n* = 6). All data are presented as mean ± SEM.

To investigate the crucial biological role of SHEP1 on regulation of cardiac function and cardiac injury under the condition of MIRI in vivo, LysM Cre^+^ SHEP1^fl/fl^ (CKO) and SHEP1^fl/fl^ (flox) littermates were generated by using gene-editing technology and then underwent the operation of IR. Echocardiography was performed to observe the difference among various groups. The data showed that despite similar baseline echocardiographic parameters, macrophage-specific deficiency of SHEP1 group resulted in worse cardiac function both 4 days and 21 days after MIRI, with a significant reduction in the ejection fraction (EF%) and fractional shortening (FS%), compared with the control group (Fig. 6A). Subsequently, the pathologic character of myocardial injury was evaluated, according to infarct area/ areas at risk (AAR) ratio and myocardial apoptosis markers. The findings displayed that AAR ratio in CKO mice with SHEP1 deficiency in macrophages was obviously higher than that in control group (Fig. 6B). Similar results were confirmed by further evidence that the increased TUNEL^+^ cardiomyocytes (Fig. 6C) in CKO mice group were more obvious, concurrently, higher expression of pro-apoptotic molecule BAX and lower expression of anti-apoptosis molecule Bcl-2 (Fig. 6D) in LV tissue samples could be observed in CKO mice group 24 h after MIRI, compared with the control group.

Additionally, myocardial fibrosis post myocardial infarction is secondary pathological changes, which could be evaluated by Masson’s trichrome staining. The results showed that myocardial fibrosis 21 days after MIRI increased significantly in the CKO group, compared with the control group (Fig. 6E). Although the increase in SHEP1 expression in macrophages after MIRI returned to baseline levels within 3 days, the reason why the difference between various groups was still significant might be due to the reflection of the integrated effect of the lack of SHEP1, the early inflammatory and myocardial injury.

### Macrophage-specific deficiency of SHEP1 exacerbates cardiac injury through amplifying inflammatory process under MIRI in vivo

**Fig. 7:**
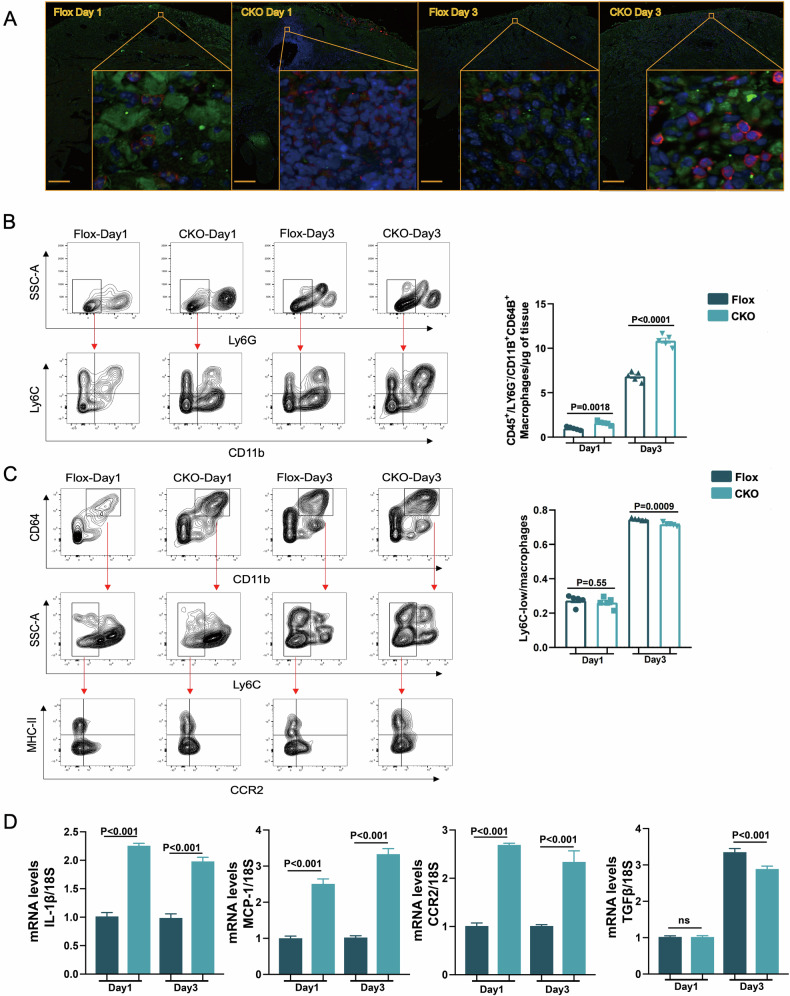
Macrophage-specific deficiency of SHEP1 exacerbates cardiac injury through amplifying inflammatory process under MIRI in vivo. **A** Representative dual-immunofluorescence staining of SHEP1 (green), F4/80 (red), and nuclei (4,6-diamidino-2-phenylindole, blue) in mice hearts (flox and CKO) 1 and 3 days after IR, F4/80^+^ (red) cell numbers were counted and compared (*n* = 4; scale bar, 500 μm). **B**, **C** Flow cytometry analysis for cardiac macrophages within LV myocardium in *LysM Cre*^*+*^ *Shep1*^*fl/fl*^ and *Shep1*^*fl/fl*^ mice 1 or 3 days after MIRI. *n* = 5 mice/group pooled from 5 independent experiments. **D** RT-qPCR analysis of the mRNA levels of inflammatory mediators in cells sorted by F4/80 magnetic beads from total cardiac cells 1 or 3 days after MIRI from *LysM Cre*^*+*^ *Shep1*^*fl/fl*^ mice and their *Shep1*^*fl/fl*^ littermates. All data are presented as mean ± SEM.

As an important source of inflammatory cytokines, macrophages play a critical role in the regulation of inflammation caused by myocardial injury. To investigate the role of SHEP1 in macrophage-mediated inflammatory response, the operation of IR was conducted in both CKO mice and flox littermates. The results indicated that by the comparison of their differences at time points of 1 and 3 days after the initiation of reperfusion, immunofluorescence staining of the myocardium in CKO mice appeared obviously enhanced infiltration of macrophages into the myocardium, compared with that in flox littermates (Fig. 7A).

Next, we used flow cytometry to evaluate the infiltration of cardiac macrophages/monocytes in the SHEP1 KO and WT mice after MIRI. The number of CD11b/CD46 double labeled positive macrophages in CKO mice hearts was significantly higher than that of control group whether 1 day or 3 days after MIRI (Fig. 7B and Supplementary Fig. 1). Besides, the proportion of Ly6c^low^/macrophages among various groups showed no remarkable difference (Fig. 7B).In addition, the CKO mice showed an increased infiltration of MHC-II^high^ CCR2^+^ macrophages and an enhanced recruitment of MHC-II^low^ CCR2^+^ monocytes after MIRI compared with the WT mice, suggesting that SHEP1 deficiency resulted in prolonged inflammation(Fig. 7C). These above flow cytometry results implied the positive role of SHEP1-deficiency improving macrophage differentiation under MIRI.

Inflammatory cytokines are important regulators of inflammatory reaction under the condition of various injuries. We further conducted magnetic cell sorting for F4/80^+^ cells from total cardiac cells of different groups of mice at 1 and 3 days after MIRI and then applied RNA analysis of the isolated cells to quantify their pro-inflammatory phenotype. The results showed that compared to the control group, the pro-inflammatory factors IL-1β and MCP-1 were significantly enriched in the CKO mice group at 1 and 3 days after MIRI. And the similar expression trend was consistent with C-C motif chemokine receptor 2 (CCR2), whereas inconsistent with TGF-β (Fig. 7D).

### Epigallocatechin gallate, an inhibitor of G3BP1, improves cardiac function of myeloid-specific SHEP1 deficiency mice under MIRI

**Fig. 8:**
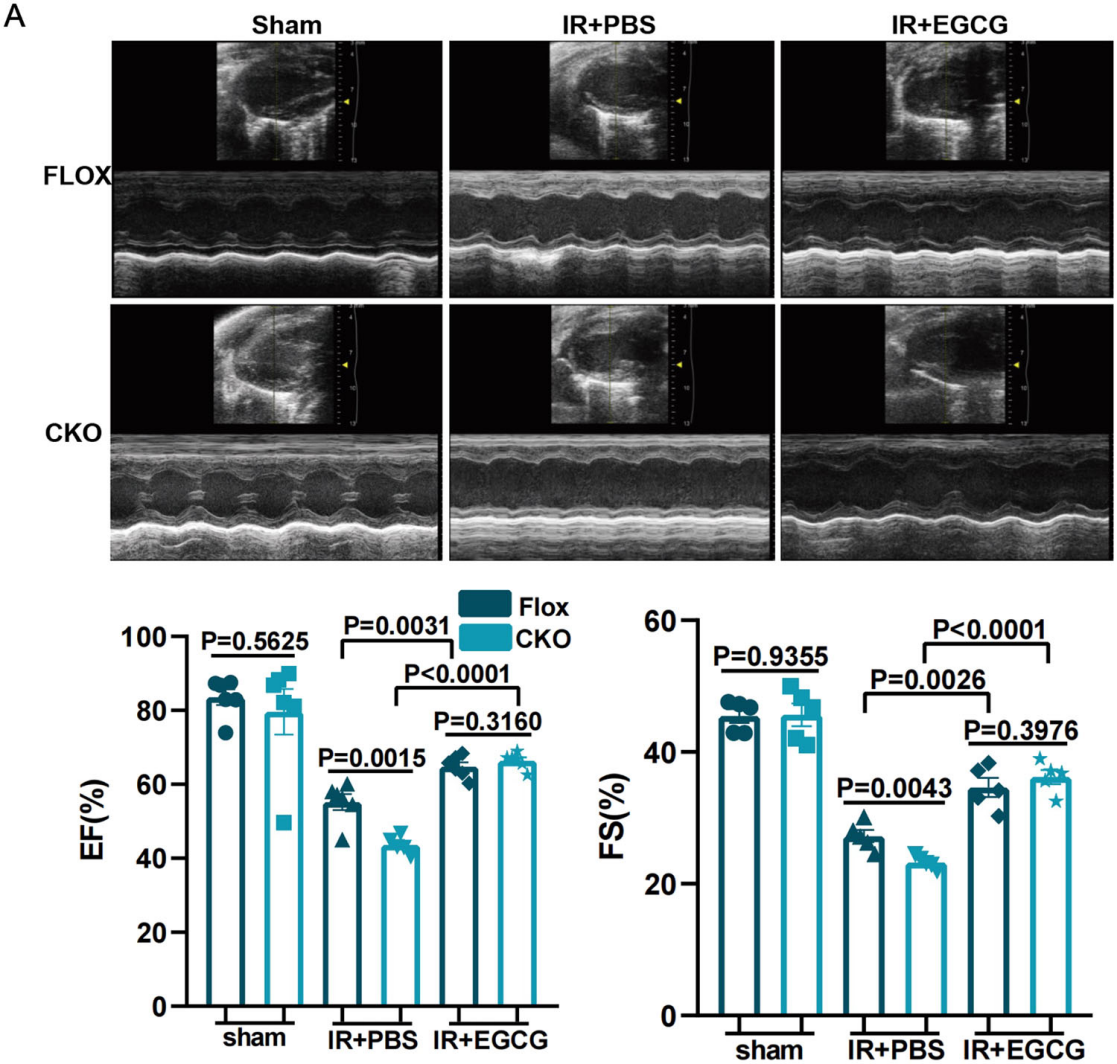
G3BP1 inhibition mproves cardiac function of myeloid-specific SHEP1 deficiency mice under MIRI. **A** *LysM Cre*^*+*^ *Shep1*^*fl/fl*^ mice and their *Shep1*^*fl/fl*^ littermates were intraperitoneally infused with EGCG or PBS as control right after IR. Echocardiography was conducted 3 days later, and representative images are displayed with quantification of percentage ejection fraction (% EF) and percentage fractional shortening (% FS) (*n* = 6). All data are presented as mean ± SEM.

To verify whether SHEP1 also competitively bound to G3BP1 to improve heart function in vivo, we first selected epigallocatechin gallate (EGCG), an inhibitor of G3BP1 extracted from green tea, as targeting reagent, and then administered it to the MIRI model mice once daily, intraperitoneally for 3 days. The results indicated that a significant improvement in cardiac function in CKO mice group receiving EGCG occurred, with increased ejection fraction (EF%) and fractional shortening (FS%), compared with that in CKO mice group receiving PBS. What’s more, the increasing extent in the above two parameters in CKO mice group was significantly higher than those in Flox mice group receiving EGCG and PBS (Fig. 8A).

## Discussion

In the course of myocardial ischemia-reperfusion injury, large quantities of monocytes invade the heart within 24 h as the dominant immune cells obliterate debris [18]; however, excessive inflammation is thought to exacerbate adverse remodeling and heart failure pathogenesis. This highlights the need for an appropriate extent of anti-inflammatory approaches within the desired time window to effectively suppress injurious processes without negatively interfering with the reparative response. However, little is known about the variety of monocytes and monocyte-derived macrophages recruited to the heart during MIRI, or the mechanisms that modulate monocyte recruitment and fate determination. In our model of clinically relevant MIRI, we observed an increased level of SHEP1 in the myocardium at early time points after injury, with subtotal expression in F4/80^+^ macrophages. Using myeloid cell-specific SHEP1 knockout mice, we demonstrated for the first time that genetic depletion of SHEP1 in macrophages exacerbated MIRI through enhanced infiltration and pro-inflammatory differentiation of monocytes, revealing a role for SHEP1 in macrophages during MIRI, implying that targeting SHEP1 on macrophages may constitute a therapeutic approach for the prevention of IR injury.

As mentioned above, SHEP1 was only highly expressed in the early stage of MIRI and decreased to baseline levels within 3 days. The early expression of SHEP1 could counteract excessive macrophage infiltration and inflammatory factor secretion only at the early stages of MIRI to further reduce myocardial tissue damage and cardiac function deterioration caused by an exaggerated inflammatory response, without directly impeding the reparative function of macrophages in the middle and late stages of injury. This makes it an ideal target and research direction for MIRI therapy.

Specialized receptors on the cell surface transduce extracellular stimuli and initiate signaling cascades that culminate in diverse functional outcomes. This organization is achieved through specific high-affinity protein-protein interactions and multiprotein complex formation facilitated by conserved modular domains. Src homology region 2 (SH2) is one of the most prevalent conserved modular domains [8]. Accordingly, we assumed that SHEP1 manipulates macrophage functionality through protein-protein interactions, and subsequently conducted protein mass spectrometry in the immunoprecipitates of SHEP1. G3BP1 was screened from those identified proteins because it can link SHEP1 to both the migration and inflammation pathways.

G3BP1 was initially discovered as a binding protein of the Ras-GTPase-activating protein (RAS-GAP), which is an inactivator of RAS GTPase via hydrolysis of the Ras-bound GTP molecule to GDP [19, 20]. With no enzymatic activity, G3BP1 acts on the RAS signaling pathway by interacting with GAP [21]. Here, we demonstrated the combination of SHEP1 and G3BP1 to a greater extent when co-cultured with hypoxia-reoxygenated cardiomyocytes, indicating a potential role of SHEP1 in RAS signaling pathway manipulation. This was confirmed by RNA-sequencing of RAW264.7, co-cultured with hypoxia-reoxygenated cardiomyocytes, and further confirmed by protein analysis, in which SHEP1 suppressed phosphorylation of ERK1/2 as well as expression of WAVE2, an activator of the Arp2/3 actin nucleator essential for cell migration through actin assembly [22]. Further blocking of G3BP1 by siRNA abolished SHEP1 influence on WAVE2, confirming that SHEP1 acts on the RAS-MAPK-ERK pathway through G3BP1.

Additionally, recent studies have revealed that G3BP1 abrogates cGAS stimulation, subsequently activating NF-κB by enhancing cGAS-DNA association independent of SGs [16, 17], despite its essential role in promoting the formation of SGs [23–26]. As a cytosolic DNA sensor, cGAS is activated robustly in myocardial infarction, triggering downstream events mediated by the STING cascade. This, in turn, promotes macrophage transformation toward the M1-like subtype [27]. Our study confirmed the G3BP1-cGAS combination, ulteriorly disclosing that it was compromised when co-cultured with H/R cardiomyocytes, consistent with the dissociation of G3BP1 from cGAS upon stimulation by DNA as revealed in previous studies. In contrast, the SHEP1-G3BP1 combination escalated resulting from an increase in SHEP1 expression. Together with a lack of direct combination detected between SHEP1 and cGAS, we concluded that SHEP1 might compete with cGAS in binding with G3BP1 to suppress cGAS activation. This competitive binding relationship needs to be confirmed by further molecular protein experiments. Therefore, SHEP1 reduces the binding of cGAS to G3BP1 by binding to G3BP1, thereby inhibiting DNA sensing of cGAS and further inhibiting the activation of its downstream cGAS-STING pathway. Subsequent assays of the phosphorylation levels of TBK1, a key molecule in the cGAS-STING pathway, validated this inference. Further blockage of either G3BP1 or cGAS abolished SHEP1 influence on NFκB, confirming that SHEP1 influences NFκB activation via the G3BP1 forward cGAS-STING pathway.

In summary, our findings reveal that during MIRI, SHEP1 expression levels in macrophages infiltrating injured myocardial tissues increased significantly in the early stages and returned to baseline levels within 3 days. It competitively bound to G3BP1, further inhibiting the activation of the RAS-MAPK-ERK pathway to suppress cell migration while inhibiting the activation of the cGAS-STING pathway to reduce the production of pro-inflammatory factors. The specific knockout of SHEP1 in macrophages promoted the infiltration of macrophages and differentiation to a pro-inflammatory phenotype during MIRI, thereby exacerbating myocardial injury and deterioration of cardiac function. Pharmacological inhibition of G3BP1 in IR-injured mice reversed the deterioration of cardiac function due to macrophage SHEP1 deficiency. Given that SHEP1 is only highly expressed at the early stage of MIRI and regulates macrophage migration and pro-inflammatory phenotype, it provides a therapeutic target and research idea for MIRI to suppress excessive inflammation only at this early stage of injury without affecting the later reparative function of macrophages.

## Supplementary information

SHEP1 alleviates cardiac ischemia-reperfusion injury via targeting G3BP1 to regulate macrophage infiltration and inflammation

Supplementary Figure 1

Supplemental Tables
