# Supplementary material for: SHEP1 alleviates cardiac ischemia reperfusion injury via targeting G3BP1 to regulate macrophage infiltration and inflammation
Source: Cell Death Dis. 2024 Dec 18;15(12):916. doi: 10.1038/s41419-024-07282-5 (PMC11655884; doi:10.1038/s41419-024-07282-5)
Supplement: Supplementary file 1 — SHEP1 alleviates cardiac ischemia-reperfusion injury via targeting G3BP1 to regulate macrophage infiltration and inflammation [file 41419_2024_7282_MOESM1_ESM.pdf]

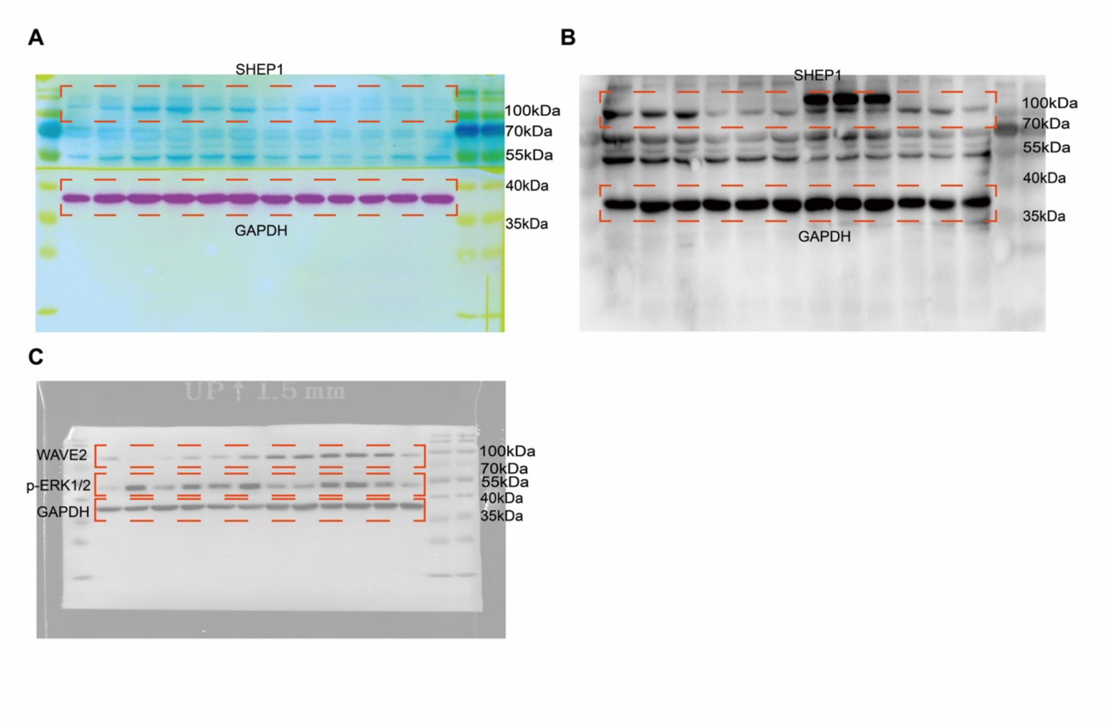

Fig. 1 A. The full uncropped western blots of Figure 1D. B. The full uncropped western blots of Figure 2B. C. The full uncropped western blots of Figure 3B.

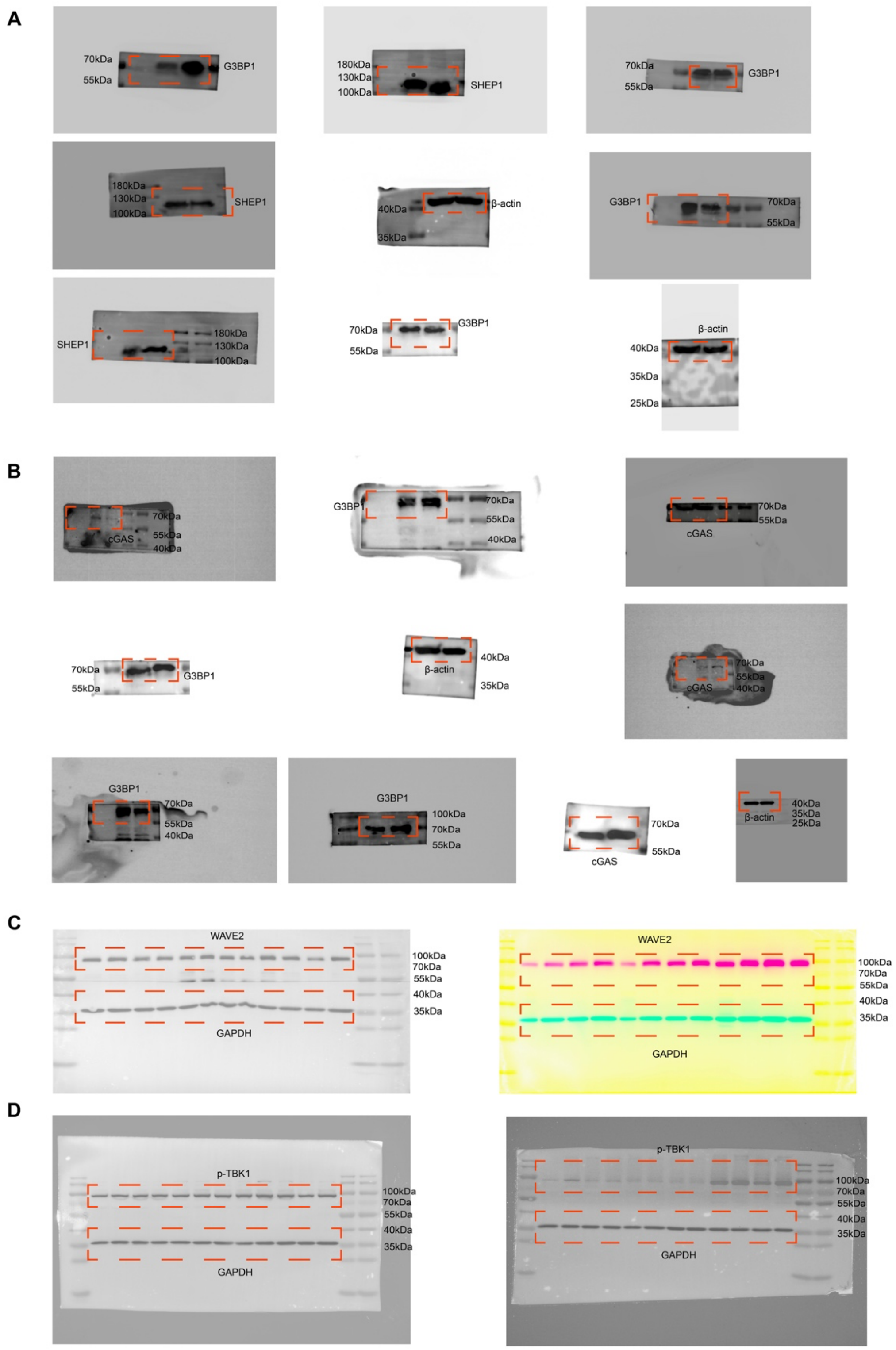

Fig. 2 A. The full uncropped western blots of Figure 4B. B. The full uncropped western blots of Figure 4C. C. The full uncropped western blots of Figure 4D. D. The full uncropped western blots of Figure 4E.

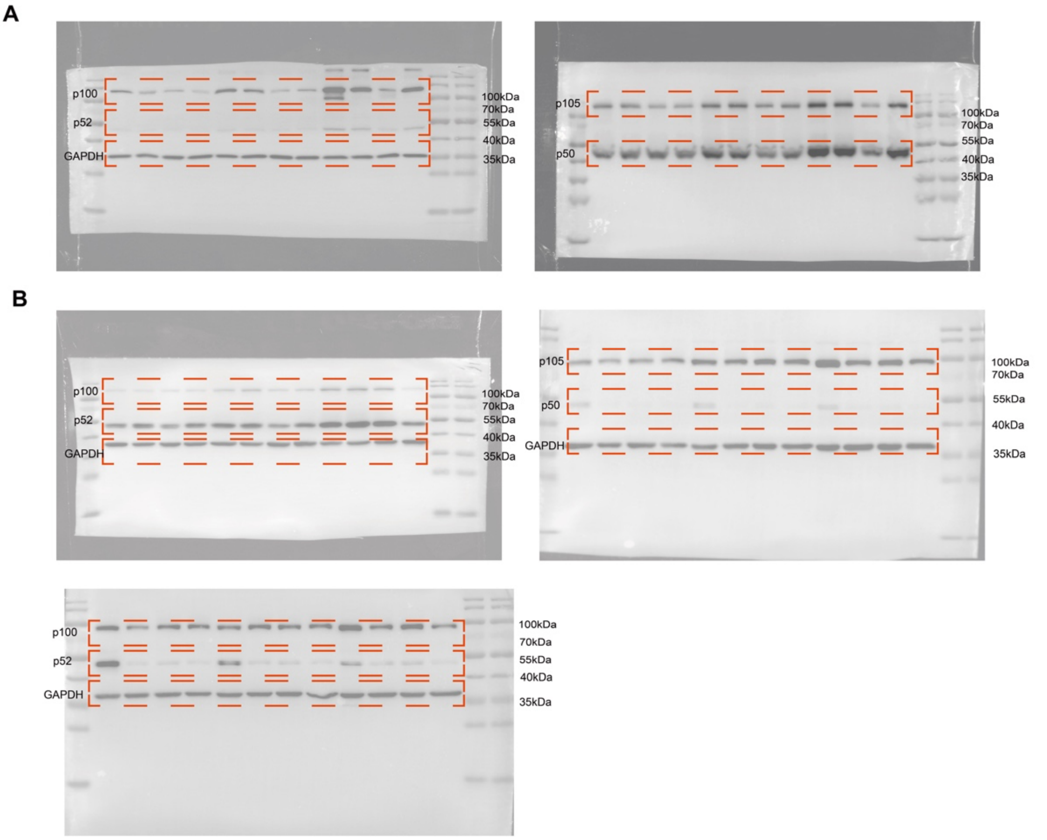

Fig. 3 A. The full uncropped western blots of Figure 4F. B. The full uncropped western blots of Figure 4G.

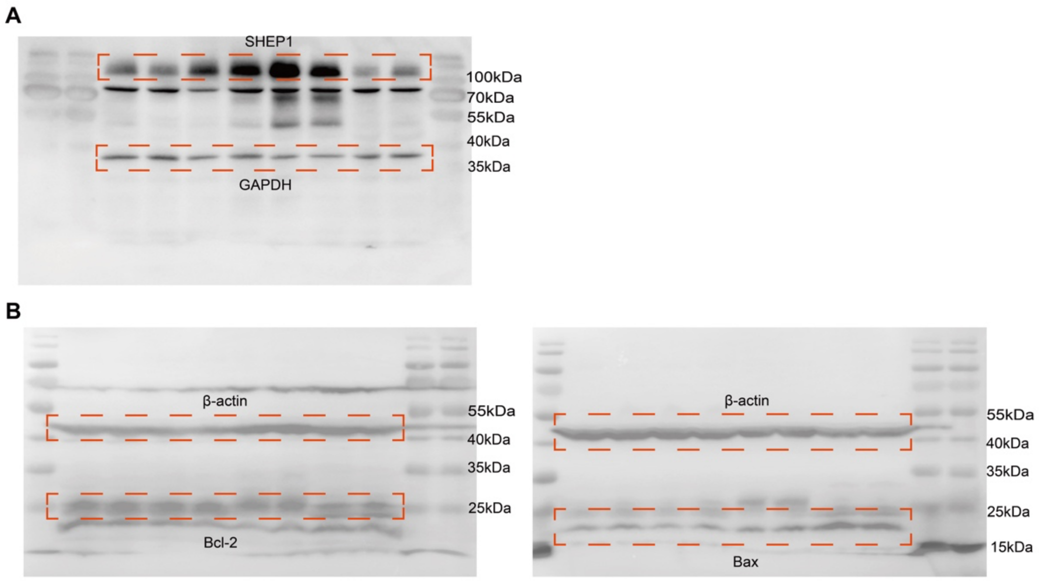

Fig. 4 A. The full uncropped western blots of Figure 5D. B. The full uncropped western blots of Figure 6D.

Target	Sample	Ctrl	Expression	Expression SEM	Corrected Expression SEM	Mean Cq	Cq SEM
18s	1					10.55	0.08028
18s	10					9.36	0.27018
18s	11					9.33	0.29443
18s	12					9.84	0.19725
18s	13					10.06	0.01417
18s	14					9.19	0.09371
18s	15					9.73	0.06678
18s	16					9.57	0.06734
18s	17					9.96	0.01094
18s	18					9.72	0.16174
18s	19					9.42	0.04614
18s	2					10.57	0.22388
18s	20					9.31	0.05465
18s	21					9.22	0.02057
18s	3					9.95	0.22507
18s	4					10.49	0.17572
18s	5					10.33	0.20525
18s	6					10.60	0.25444
18s	7					9.86	0.22451
18s	8					10.05	0.23551
18s	9					10.26	0.21468
sh	1		1.61894	0.16780	0.16780	31.15	0.12616
sh	10		0.83945	0.16663	0.16663	30.91	0.09497
sh	11		1.01940	0.21140	0.21140	30.60	0.05312
sh	12		1.08983	0.17171	0.17171	31.02	0.11296
sh	13		0.90608	0.01219	0.01219	31.50	0.01326
sh	14		0.54443	0.03558	0.03558	31.36	0.01036
sh	15		0.63829	0.06977	0.06977	31.67	0.14286
sh	16		0.74869	0.06776	0.06776	31.28	0.11186
sh	17		1.00593	0.03943	0.03943	31.24	0.05549
sh	18		1.11349	0.12724	0.12724	30.86	0.03190
sh	19		0.67886	0.02218	0.02218	31.28	0.00960
sh	2		1.69711	0.26388	0.26388	31.10	0.01411
sh	20		0.52303	0.02587	0.02587	31.54	0.04589
sh	21		0.53094	0.05232	0.05232	31.43	0.14068
sh	3		1.12755	0.20725	0.20725	31.07	0.14021
sh	4		2.45101	0.31549	0.31549	30.50	0.06007
sh	5		2.20451	0.54834	0.54834	30.48	0.29435
sh	6		2.62969	0.53229	0.53229	30.50	0.14330
sh	7		1.20064	0.18719	0.18719	30.89	0.01376
sh	8		1.36098	0.24046	0.24046	30.90	0.09750
sh	9		1.00864	0.15459	0.15459	31.55	0.05295

Fig. 5 The raw qPCR data of Fig 1C.

Target	Sample	Ctrl	Expression	Expression SEM	Corrected Expression SEM	Mean Cq	Cq SEM
Actin	CON1					14.82	0.04106
Actin	CON2					14.88	0.05874
Actin	CON3					14.74	0.06555
Actin	KO1					15.27	0.06036
Actin	KO2					14.87	0.06120
Actin	KO3					14.98	0.07535
Actin	OE1					14.53	0.02365
Actin	OE2					14.69	0.03348
Actin	OE3					14.59	0.05151
Actin	WT1					14.73	0.05033
Actin	WT2					14.68	0.04275
Actin	WT3					14.70	0.03594
SH	CON1		0.12575	0.00634	0.00634	32.44	0.06003
SH	CON2		0.14296	0.00984	0.00984	32.31	0.08008
SH	CON3		0.14661	0.00807	0.00807	32.14	0.04484
SH	KO1		0.00256	0.00069	0.00069	38.50	0.38318
SH	KO2		0.00315	0.00110	0.00110	37.80	0.49776
SH	KO3		0.00565	0.00146	0.00146	37.08	0.36531
SH	OE1		0.98887	0.02083	0.02083	29.18	0.01908
SH	OE2		0.96162	0.03629	0.03629	29.37	0.04294
SH	OE3		1.04321	0.04810	0.04810	29.16	0.04209
SH	WT1		0.44820	0.02789	0.02789	30.52	0.07435
SH	WT2		0.41939	0.01530	0.01530	30.56	0.03069
SH	WT3		0.37794	0.01835	0.01835	30.73	0.06014

Fig. 6 The raw qPCR data of Fig 2A.

Target	Sample	Ctrl	Expression	Expression SEM	Corrected Expression SEM	Mean Cq	Cq SEM
18S	C0					6.85	0.21931
18S	C1					6.98	0.04373
18S	C3					7.31	0.16809
18S	C6					7.19	0.06568
18S	F0					7.11	0.03993
18S	F1					7.08	0.02467
18S	F3					8.20	0.11104
18S	F6					7.35	0.05209
A	C0		0.00036	0.00007	0.00007	39.20	0.01036
A	C1		0.15705	0.01614	0.01614	30.54	0.14167
A	C3		1.37606	0.18906	0.18906	27.74	0.10505
A	C6		0.95414	0.04578	0.04578	28.15	0.02185
A	F0		0.00101	0.00005	0.00005	37.94	0.00000
A	F1		0.00286	0.00114	0.00114	36.43	0.57649
A	F3		1.61849	0.13408	0.13408	28.40	0.04421
A	F6		1.17158	0.04439	0.04439	28.02	0.01658
B	C0		0.00578	0.00088	0.00088	29.12	0.01973
B	C1		1.09082	0.04446	0.04446	21.69	0.03931
B	C3		0.49508	0.06027	0.06027	23.16	0.05094
B	C6		0.11232	0.00552	0.00552	25.18	0.02685
B	F0		0.01448	0.00062	0.00062	28.05	0.04691
B	F1		0.32937	0.01709	0.01709	23.52	0.07065
B	F3		0.09204	0.00918	0.00918	26.48	0.09143
B	F6		0.26129	0.01369	0.01369	24.12	0.05478
C	C0		0.01826	0.00329	0.00329	33.11	0.13941
C	C1		1.09082	0.03915	0.03915	27.33	0.02774
C	C3		1.35719	0.16852	0.16852	27.35	0.06192
C	C6		0.25925	0.01207	0.01207	29.62	0.01407
C	F0		0.03606	0.00142	0.00142	32.38	0.04061
C	F1		0.26071	0.01420	0.01420	29.51	0.07462
C	F3		0.32723	0.03266	0.03266	30.29	0.09167
C	F6		1.06852	0.04114	0.04114	27.74	0.01930

Target	Sample	Ctrl	Expression	Expression SEM	Corrected Expression SEM	Mean Cq	Cq SEM
18S	C0					7.54	0.07215
18S	C1					7.39	0.01862
18S	C3					8.00	0.11643
18S	C6					8.29	0.02418
18S	F0					7.99	0.07897
18S	F1					7.10	0.05264
18S	F3					8.43	0.07444
18S	F6					7.55	0.12269
A	C0		0.01399	0.00091	0.00091	30.75	0.06062
A	C1		1.22736	0.02401	0.02401	24.15	0.02121
A	C3		0.59864	0.05005	0.05005	25.79	0.03150
A	C6		0.08541	0.00352	0.00352	28.89	0.05435
A	F0		0.01997	0.00182	0.00182	30.69	0.10487
A	F1		0.41431	0.01765	0.01765	25.42	0.03174
A	F3		0.14071	0.01063	0.01063	28.31	0.07966
A	F6		0.24177	0.02260	0.02260	26.65	0.05590
B	C0		0.22520	0.01633	0.01633	33.89	0.07574
B	C1		1.22736	0.02303	0.02303	31.30	0.01965
B	C3		1.15569	0.11585	0.11585	32.00	0.08580
B	C6		0.66432	0.01781	0.01781	33.09	0.03020
B	F0		0.23190	0.01476	0.01476	34.30	0.04686
B	F1		0.38676	0.02089	0.02089	32.67	0.05746
B	F3		0.62761	0.04668	0.04668	33.31	0.07729
B	F6		0.68909	0.06485	0.06485	32.29	0.05816
C	C0		0.00976	0.00182	0.00182	33.53	0.25898
C	C1		0.36127	0.01092	0.01092	28.18	0.03943
C	C3		1.87107	0.15414	0.15414	26.42	0.02390
C	C6		0.41667	0.00912	0.00912	28.87	0.02029
C	F0		0.02192	0.00227	0.00227	32.82	0.12673
C	F1		0.16861	0.01082	0.01082	28.98	0.07616
C	F3		0.42785	0.02780	0.02780	28.98	0.05696
C	F6		0.82014	0.07843	0.07843	27.16	0.06309

Target	Sample	Ctrl	Expressio n	Expressio n SEM	Corrected Expressio n SEM	Mean Cq	Cq SEM
18S	C0					7.74	0.03794
18S	C1					7.52	0.07123
18S	C3					8.20	0.03921
18S	C6					8.41	0.08799
18S	F0					7.95	0.12274
18S	F1					7.54	0.11452
18S	F3					8.70	0.11769
18S	F6					7.73	0.12531
A	C0		0.00274	0.00051	0.00051	38.72	0.26610
A	C1		0.21142	0.01722	0.01722	32.23	0.09348
A	C3		1.60464	0.06556	0.06556	29.98	0.04402
A	C6		1.30646	0.10896	0.10896	30.49	0.08207
A	F0		0.01890	0.00190	0.00190	36.14	0.07697
A	F1		0.03199	0.00323	0.00323	34.97	0.09014
A	F3		1.29370	0.14266	0.14266	30.79	0.10705
A	F6		0.92562	0.09672	0.09672	30.30	0.08379
B	C0		0.24155	0.00649	0.00649	34.18	0.00803
B	C1		1.00000	0.04974	0.04974	31.91	0.00869
B	C3		1.20450	0.12851	0.12851	32.33	0.14884
B	C6		0.65255	0.04537	0.04537	33.42	0.04815
B	F0		0.22544	0.02731	0.02731	34.49	0.12441
B	F1		0.44338	0.04876	0.04876	33.11	0.10982
B	F3		0.74316	0.08351	0.08351	33.52	0.11149
B	F6		0.82343	0.07195	0.07195	32.40	0.01372
C	C0		0.00935	0.00097	0.00097	32.85	0.14426
C	C1		0.23844	0.01374	0.01374	27.95	0.04293
C	C3		1.60464	0.04410	0.04410	25.88	0.00587
C	C6		0.24978	0.03547	0.03547	28.78	0.18502
C	F0		0.01173	0.00342	0.00342	32.73	0.40174
C	F1		0.15959	0.01630	0.01630	28.55	0.09269
C	F3		0.26966	0.02425	0.02425	28.95	0.05458
C	F6		0.53074	0.04614	0.04614	27.00	0.00528

Target	Sample	Ctrl	Expression	Expression SEM	Corrected Expression SEM	Mean Cq	Cq SEM
18S	C0					8.98	0.04117
18S	C1					8.83	0.08431
18S	C3					9.09	0.19121
18S	C6					9.53	0.16168
18S	F0					9.07	0.02792
18S	F1					8.54	0.11192
18S	F3					9.74	0.15999
18S	F6					8.95	0.07033
A	C0		0.00751	0.00045	0.00045	29.21	0.07539
A	C1		1.22233	0.08316	0.08316	21.71	0.05025
A	C3		0.50656	0.06771	0.06771	23.25	0.02504
A	C6		0.17130	0.01932	0.01932	25.26	0.01847
A	F0		0.01664	0.00058	0.00058	28.16	0.04144
A	F1		0.26876	0.02301	0.02301	23.61	0.05220
A	F3		0.08405	0.00950	0.00950	26.49	0.03123
A	F6		0.26125	0.01569	0.01569	24.06	0.05059
B	C0		0.02719	0.00323	0.00323	34.73	0.16623
B	C1		1.22233	0.07913	0.07913	29.09	0.04018
B	C3		1.11235	0.16094	0.16094	29.49	0.08374
B	C6		0.27361	0.03183	0.03183	31.96	0.04507
B	F0		0.02640	0.00231	0.00231	34.87	0.12301
B	F1		0.26908	0.03255	0.03255	30.98	0.13391
B	F3		0.32263	0.04475	0.04475	31.92	0.12022
B	F6		1.26674	0.08350	0.08350	29.16	0.06401
C	C0		0.11211	0.01313	0.01313	29.80	0.16383
C	C1		1.14777	0.06832	0.06832	26.30	0.01632
C	C3		1.46882	0.19731	0.19731	26.21	0.03161
C	C6		1.13736	0.13041	0.13041	27.02	0.03498
C	F0		0.17118	0.00380	0.00380	29.29	0.01566
C	F1		0.38539	0.04203	0.04203	27.59	0.11060
C	F3		0.86253	0.10156	0.10156	27.62	0.05711
C	F6		1.32972	0.07478	0.07478	26.21	0.04046

Fig. 7 The raw qPCR data of Fig 7D.
