# Supplementary material for: SHEP1 alleviates cardiac ischemia reperfusion injury via targeting G3BP1 to regulate macrophage infiltration and inflammation
Source: Cell Death Dis. 2024 Dec 18;15(12):916. doi: 10.1038/s41419-024-07282-5 (PMC11655884; doi:10.1038/s41419-024-07282-5)
Supplement: Supplementary file 3 — Supplemental Tables [file 41419_2024_7282_MOESM3_ESM.docx]

**SHEP1 Antagonizes Cardiac Ischemia-reperfusion Injury by Regulating Macrophage Infiltration and Inflammation**

**Online Resources**

**Supplementary Table 1.** **Primers for RT-qPCR**

| **Gene** | **Forward primers (5’- 3’)** | **Reverse primers (5’- 3’)** |
| --- | --- | --- |
| 18S | CGGCTACCACATCCAAGGAA | CCTGTATTGTTATTTTTCGTCACTACCT |
| Sh2d3c | CTAGAGCCCCGAAGGACTG | GGCGAGGAGTCAAGAATGTACTT |
| IFN-β | TGGGTGGAATGAGACTATTGTTG | CTCCCACGTCAATCTTTCCTC |
| IL-1β | GCAACTGTTCCTGAACTCAACT | ATCTTTTGGGGTCCGTCAACT |
| MCP-1 | TTAAAAACCTGGATCGGAACCAA | GCATTAGCTTCAGATTTACGGGT |
| CCR2 | ATCCACGGCATACTATCAACATC | CAAGGCTCACCATCATCGTAG |
| TGF-β1 | CTCCCGTGGCTTCTAGTGC | GCCTTAGTTTGGACAGGATCTG |

**Supplementary Table 2 Antibodies Used in Western-blot**

| **Antibody specificity** | **Dilution** | **Company** | **Cat. No.** |
| --- | --- | --- | --- |
| **SHEP1** | 1:1000 | Santa Cruz | sc-100792 |
| **Gapdh** | 1:4000 | Invitrogen | 437000 |
| **β-Actin** | 1:4000 | CST | 3700S |
| **BAX** | 1:1000 | CST | 14796S |
| **Bcl-2** | 1:1000 | CST | 3498S |
| **WAVE2** | 1:1000 | CST | 3659S |
| **TBK1** | 1:1000 | CST | 38066S |
| **p-TBK1** | 1:1000 | CST | 5483S |
| **ERK1/2** | 1:1000 | CST | 9102S |
| **p-ERK1/2** | 1:1000 | CST | 9101S |
| **P100/52** | 1:1000 | CST | 4882S |
| **P105/50** | 1:1000 | CST | 12540S |
| **G3BP1** | 1:1000 | CST | 61559S |
| **cGAS** | 1:1000 | CST | 31659S |
